# Collectanea Medica, Consisting of Anecdotes, Facts, Extracts, Illustrations, Queries, Suggestions, &c.

**Published:** 1816-09

**Authors:** 


### COLLECTANEA MEDICA,

CONSISTING OF

### ANECDOTES, FACTS, EXTRACTS, ILLUSTRATIONS, QUERIES, SUGGESTIONS, &c.

RELATING TO THE

*History or the Art of Medicine, and the Auxiliary Sciences.*

Quicquid agunt medici,

Nostri farrago libelli.

SOME of our readers, who indulge in miscellaneous reading, may have met with notes on the sea long-worm in the Memoirs lately published of the late Mr. Holcroft's Life. They were, however, entirely conjectural. The following, from the last number published by the Linnæan Society, and from so respectable a member, are deserving of all the credit due to human evidence.

" *Some Observations on the Sea Long-Worm of Borlase,* Gordius marinus *of Montagu.* By the Rev. HUGH DAVIES, F.L.S.

" As the concise definition of the genus *Gordius*, in the *Systema Naturæ*, does by no means include the characters of this very extraordinary animal, I deem it necessary to give the following more comprehensive one of it under the other name which has been bestowed on it.

" LINEUS longissimus. *Sowerby's Brit. Misc.* p. 15, t. viii.

" *Black Line-worm.*

" CORPUS lineare, lævissimum, longissimum, mirandum in modum exertile et retractile.

" CAPUT antice emarginatum, proboscidem cylindrico-clavatam exserens.

" OS inferum, lineare, longitudinale.

" OCULI nulli.

" This is the *Gordius marinus* of Mr. Montagu, but not *Gordius marinus* of Linnæus.

" I laid a perfect specimen of this very wonderful creature in its own element in the largest dish I have, with a design to observe its habits or manners.

" It partook in a great measure of the nature of the leech, and seemed in some degree amphibious; as it frequently, in part, left the water, and, to the length of a foot or two, or more, extended itself along the edge of the dish, and the table on which the dish was placed. At other times, particularly in the day-time, it was compactly collected together in a heap, and perfectly still, unless the dish or table was touched, of which it seemed very sensible. This it indicated by a vibratory motion of its whole mass, and retracting retracting the head and forepart, which were generally somewhat extended. In the night I always found it coiled in a more lax and diffuse manner, covering nearly the whole dish; but on the approach of a candle it seemed affected, and inclined to contract itself; so that, although I could not see that it had eyes, I evidently discerned that it was very sensible of light. It frequently by morning assumed somewhat of a spiral or screw-like form; and on one morning in particular I was highly gratified in finding it almost perfectly and closely spiral from end to end. I was forcibly struck with this appearance, as it seemed to suggest to me the solution of a difficulty which perplexed me much, concerning the manner how such a wonderfully soft, delicate, and seemingly unmanageable length of body could possibly move itself from one place to another. But from the moment when I observed this, I became perfectly at ease with regard to that particular, being convinced that this must be the state which the creature assumes when disposed to change its station; not only as it is thus contracted, with regard to length, into the most compact size of which its make is susceptible, but likewise that, when so modified, every spire or volution, by a distinct impulse exerted in an appropriate manner, will assist in the act of progression, and of shifting forward the whole of its amazing length at nearly the same instant.

" When I took it up at the sea-side, collecting such an immense length in a confused manner into the confined space of an oyster-shell, (a very large one indeed,) I thought it had been almost impossible to have unravelled it; but it is astonishing to think how readily it was disentangled, owing to the extraordinary profusion of mucus which nature has provided it with, doubtless for that purpose.

" It is impossible to make a guess at the length of it when alive, on account of its constantly extending and contracting itself when touched, and that with such ease as almost to exceed belief. I once observed a part of the fore end extended to a length between two and three feet, along the margin of the dish and the table, which part, on the animal being disturbed, was in a short time contracted, so as not to exceed so many inches; and, as I assert that, when it was thus extended, it was full three times the size in diameter which I had seen it on some other occasions, I may well say that it is capable of extending itself, or of being extended, without any inconvenience, to twenty-five or thirty times the length that it is of at another time.

" It varies very considerably in colour as it contracts or extends itself, which is from a dusky to a reddish-brown; but it has, when placed in a strong light, especially in sunshine, a gloss of a fine rich purple all over; when most contracted it appears nearly black.

" Having thus attended to this remarkable animal for a fortnight, giving it daily a fresh supply of sea-water, I put it into a bottle, which, by the by, though the bottle was wide-mouthed,

I effected

I effected with no little trouble, owing to its facility of extending and contracting itself, and likewise its being so slippery from the quantity of mucus with which it abounds. When, however, this was done, I poured on it some spirits; it was convulsed, and greatly contracted with regard to length, and consequently much enlarged in thickness, though neither nearly to that degree which I had often observed when it was alive; and in an instant, to my great surprise, it projected, from the emarginate part of the front, a proboscis which was eight inches in length. It is very strange, that, during the space of time above stated, and the various treatment which the creature had experienced, as well as the different attitudes and states I had seen it in, it never in the least exhibited this part of itself till in its dying convulsion.

"It being, as I have before observed, impossible while the animal was alive to make any reasonable conjecture as to the length and breadth of it, I took it out of the bottle, and, on measuring it, found it full *two-and-twenty feet* long, exclusive of the proboscis.

"Now, after the various and repeated observations which I have made, I give it as my firm opinion, that I speak within bounds when I say the animal, when alive, might have been extended to four times, at least, its length when dead. I therefore look on what Mr. Sowerby gives, on the authority of the fishermen at Newhaven, to be by no means improbable, viz. that this most astonishing creature may have been known to be susceptible of being drawn to the length of *twelve fathoms;* or, according to the account of the fishermen on the south coast of Devonshire, to Mr. Montagu, to *thirty yards* or *fifteen fathoms.* Indeed, Mr. Montagu's own account, of one of the length of *eight feet* when alive, being reduced to *one foot* when immersed in spirits, does more than support my opinion.

"This subject and another specimen were found beneath the Green, near Beaumares, at the time of spring-tides, in the month of March 1812."

This half volume contains a very long and valuable paper from Dr. Leech, of whom it may be said, to use the language of a naturalist, *Habitat in Musæo in agris & inter insecta, &c.* As the paper occupies nearly one hundred of the quarto pages, and as many of our readers are members of that valuable society, we shall content ourselves with giving the title and general divisions, selecting afterwards a few of the most interesting articles.

"*A tabular View of the external Characters of Four Classes of Animals, which Linné arranged under* Insecta; *with the Distribution of the Genera composing Three of these Classes into Orders, &c. and Descriptions of several New Genera and Species.* By William Elford Leach, M.D.—From the same.

"The object of this paper is chiefly to call the attention of entomologists to examine into the propriety of constituting a new class to comprehend the *Syngnatha* and *Chilognatha* of Fabricius, which Latreille and Lamarck have arranged with the *Arachnides*.

"As the leading characters of the classes which were considered by Linné as Insecta are very obvious, I shall in the first place content myself with submitting to the society the external characters, through the medium of a table, and shall then consider three of the classes separately.

"All the animals in question agree in having articulated limbs for motion, and they all have their spinal mass of nerves composed of ganglia, which are formed as it were on a cord; or, in other words, are brought into communication with each other.

"By the following table the most obvious points of distinction may readily be learnt.

"A. *Branchiis pro respiratione.*

| | |
|---|---|
| "Classis I. - - - - - - - - | CRUSTACEA. |
| "B. *Tracheis pro respiratione.* | |
| "Classis II. Pedibus ultra 8. Capite distincto; antennis 2. - - - - - - | MYRIAPODA. |
| "Classis III. Pedibus 6 aut 8. Capite thoraceque coalitis; antennis 0. - - | ARACHNIDES. |
| "Classis IV. Pedibus 6. Capite distincto; antennis 2. - - - - - - - | INSECTA." |

In the first class the genus Limnoria is confined to a single species, which, as it appears new to Dr. Leach, must be so, we conceive, to most of our readers.

"*Caput* corporis segmento antico latitudine æquale. *Oculi* distincti granulati. *Cauda* segmentis plurimis corpore vix angustioribus, ultimo subrotundato.

"Antennæ infernè per paria insertæ, unâ super alteram positâ. Oculi e granulis (*octo aut septem) efformati.

"Spec. 1. *Limnoria terebrans.*

"L. corpore cinereo, oculis subpiceo-atris.

"Limnoria terebrans. *Leach, Edin. Encycl.* vii. 433.

"Long. Corp. 1 lin. et $1\frac{1}{2}$ aut 2 lin.

"This new and highly interesting species I received through the politeness of my attentive and worthy friend R. Stephenson, Esq. It occurs in the greatest abundance at the Bell Rock, in the old wood-work used whilst the lighthouse was building, which it perforates in a most alarming manner, entering to the depth of two inches or more, boring in every direction. They seldom or never deviate from a straight line in their perforations, unless interrupted in their progress by a knot in the wood, when they pass round it. The female is one-third larger than the male, and may be readily distinguished by its pouch, which is easily seen, and in which the eggs and young ones after their exclusion are carried.

"* I mention the number with some doubt; seven granules are arranged in a circle, and in a certain light there seems to be another in the centre."

The

The young ones in those I examined were generally seven in number, in some few nine, and in one instance only five. When alive it can contract nearly into a ball. I was at first induced to place it in the genus *Cymothoa*, but a more careful observation clearly proved it not to be referable to that genus."

The second class was arranged by Latreille with the arachnides.

The third class *arachnides*, contains the following well authenticated and interesting history.

"Araneides, *Latr.*

"*Oculi* sex aut octo. *Anus* papillis texoriis.

"Araneides. *Latr. Walck.*

"For the genera of this family, see *Latreille's Genera Crustaceorum et Insectorum;* and his *Considérations Générales sur l'Ordre Naturel des Crustacés, &c.*

"Having been favoured with some very valuable and highly interesting remarks on the growth of the legs of a species of this family, by that learned and indefatigable naturalist, Sir Joseph Banks, I take this opportunity of communicating them to the public.

"As Sir J. Banks was writing at Spring Grove, on the 2d of September, one of the web-spinning species, of more than the middle size, passed over some papers on the table, holding a fly in its mouth. Much surprised to see a spider of this description walking about with its prey, and struck with somewhat unusual in the gait of the animal, he caught it, and placed it in a glass for examination; when instead of eight, he perceived that it had but three legs, which accounted for the inability of the creature to spin its web. But the curious circumstance of its having changed its usual economy, and having become a hunting instead of a spinning one, as well as a wish to learn whether its legs would be renewed, induced him to keep the animal in the glass, from whence it could not escape, and to observe its conduct.

"On the following morning the animal ate two flies given to it, by sucking out the juices, but left the carcases whole. Two or three days after it devoured the body and head of a fly, leaving only the wings and legs. After this time it sometimes sucked and sometimes ate the fly given it. This probably depended on the state of the fly. At first it consumed two flies in a day, afterwards not more than one in two days. Its excrement, which it voided from the extremity of the abdomen, was at first of a milky-white colour; but afterwards the white had a black spot in the centre, of a more solid appearance than the surrounding fluid.

"Soon after its confinement it attempted to form a web on the side of the vessel, but performed the business very slowly and clumsily, from the want of the proper number of legs. In about a fortnight it had completed a very small web, upon which it generally sat.

"A month after having been caught, it shed its skin, leaving the slough hanging on the web. After this change five new legs appeared, not half as long as the other three legs, and of very little use use to the animal in walking. These new members, however, extended themselves a little in about three days, and became half as long as the old ones: the web was now increased, and the animal continued almost immoveably sitting upon it in the day-time, unless drawn from it or attracted by a fly thrown to it as its usual provision.

"Twenty-nine days afterwards it again lost its skin, leaving the slough hanging in the web, in front of a hollow cell it had woven so as to prevent it from being completely seen when lodged in it: the legs were now longer than before the change of skin, and they grew somewhat longer still in three or four days, but did not attain the size of the old legs.

"The animal now increased its web, and, being put into a small bowl as a more commodious residence, soon renewed a better web than the first. In this state it was left on the 1st of November, in the hope of being found alive in the next summer, when flies re-appear, and being subjected to further observations.

"On observing this animal, it appeared to this acute naturalist, that those organs called palpi were used by the animal in grasping and changing the position of its food whilst applied to the action of the mandibules, serving in fact the purposes of hands. Hence it occurred to Sir Joseph Banks that these parts were improperly named, and that they were really similar in function to the claws of scorpions; which opinion is firmly supported by analogy, as shall on some future occasion be shown, when the subject has undergone further examination.

"Clerk calls the *palpi*, brachia; and asserts that they contain the organs of generation; an opinion entertained also by Linné, who says '*Penes in palpis gerunt;*' but, as Sir J. Banks observes, this opinion is no where supported by a statement of facts, or of anatomical examination. That the palpi of all male spiders are clavate at their extremities, every naturalist well knows; but, if they really contain the sexual organs of the male, it is a circumstance of a most curious nature, and well worth the attentive examination of the physiologist; and we shall feel much obliged to any naturalist who can give any information as to the truth or falsity of this anomalous statement."

Such is a very brief outline of the large additions made by Dr. Leech to this branch of natural history. We confess ourselves not completely reconciled to his improved arrangement. It is not to be supposed that we should question the accuracy or judgment of such a writer; but we are not sufficiently prepared for more than classes, orders, genera, species, and varieties. Hence *subdivisio, familia subclassis*, and some others, are terms not readily accommodated to our old habits.

The succeeding article is well deserving of notice.

"*Description of a Fossil Alcyonium, from the Calk Strata near Lewes; in a Letter to A. B. Lambert, Esq. F.R.S. V.P.L.S.* By Mr. Gideon Mantell, F.L.S.—From the same.

"The *Alcyonite* which forms the subject of the present communication nication is, I believe, peculiar to the upper or flinty chalk in the vicinity of Lewes; it never occurs in any other stratum, and there is every reason to conclude that it obtains the same situation in the hills of Wiltshire. I am not aware of any author having noticed this interesting fossil, unless the funnel-shaped fossils found by M. Guettard at Nerest and Touraine, and those described in the second volume of Mr. Parkinson's admirable work on "Organic Remains," are of this species. The specimens which occur at Lewes, though generally considered as *Alcyonia*, do not entirely conform to the character of that genus as given by modern writers; yet, being evidently very nearly allied to it, the society will, perhaps, permit me to extend the character, so as to allow these fossils a temporary admission, till future discoveries shall point out more precisely their situation in the scale of animated nature.

"ALCYONIUM CHONOIDES*.

"*Funnel-like Alcyonium.*

"CHAR. GEN. Animal plantiforme, carnosum, gelatinosum, spongiosum, vel coriaceum, cellulis vel tubulis repletum. Superficies poris seu osculis hydras tentaculatas oviparas exserentibus, pertusa. Stirps fixa.

"CHAR. SPEC. A. infundibuliforme, superficie interiore tubulorum extremitates apertas exteriore fibras reticulatas exhibente.

"From an attentive examination of the mineralized remains of the *Alcyonium*, it is certain that the recent animal possessed great powers of contraction and expansion, which enabled it to assume various dissimilar forms. In a quiescent state it was more or less funnel-like, when partly expanded cyathiform, and when completely dilated it presented the figure of a broad circular disk. To this versatility of shape is to be attributed the great diversity of appearance observable in its reliquiæ, whose forms must have been derived from the contracted or expanded state of the original at the period of its introduction into the mineral kingdom. Without the knowledge of this fact, fossils originating from the same prototype are liable to be considered as distinct species, since it is by the possession of numerous specimens only that the true character of this zoophyte can be ascertained.

"That the animal enjoyed the powers of contraction and expansion above ascribed to it, will appear evident from an investigation of its structure. The epidermis, or external coat, is composed of fasciculi of muscular fibres, which, arising from the pedicle, proceed in a radiated manner toward the circumference, and, by frequently anastomosing, constitute a retiform plexus capable of dilating, lengthening, and contracting, according to the impressions it received. The fasciculi are further connected by lateral processes, which increase the firmness and coherence of the external integument. From the inner surface of the muscular envelopment arise innumerable tubuli, which pass direct to the ventricular cavity, and

* "ἀ χώνη, infundibulum.

terminate terminate in openings on its surface. In some specimens a substance of a sponge-like appearance fills up the interstices between the tubuli, and probably is the remains of a membrane, which served in the recent animal to connect the tubes and assist in strengthening and uniting the whole mass. The sides of the ventricular cavity are generally about one-third of an inch in thickness. From the basis or pedicle proceed fibres by which the animal was attached to its appropriate habitation. These facts beautifully illustrate the anatomy and physiology of the funnel-like *Alcyonium*. We find it possessing a structure, simple yet admirable, and well adapted for the purposes of its existence; an external muscular coat, which enabled it to perform its requisite motions, and a ventricular cavity with an absorbing surface, by which nutrition was effected. We have, in short, the organs which Richerand considers as characteristic of zoophytal animation. 'The zoophyte, whose name indicates an animal plant, is totally separated from all beings of the vegetable kingdom, by the existence of a cavity in which alimentary digestion is carried on; a cavity by the surface of which is an absorption, an imbibition, far more active than that which takes place by the external surface of the body.—We find a tube of soft substance, sensible and contractile in all its parts. Moisture oozes from the internal surface of the tube, softens and digests the aliments which it finds there; the whole mass draws in nourishment from it; the tube then spontaneously contracts, and casts out the residue of digestion.'—*Richerand's Physiology*, pp. 8 and 13."

---

The dispute concerning temperature in phthisis pulmonalis has, for some time past, occupied the pens of several ingenious physicians. We cannot, therefore, offer a more acceptable present than the observations of one who saw the effects of the disease in subjects circumstanced alike in all respects excepting climate.

*On Phthisis Pulmonalis;* by Sir James Macgrigor.

[From his Account of the Army in the last vol. of Med. Chir. Trans.]

In England, this disease is as frequent and fatal in the army as it is in civil life: in Spain and Portugal we found it of much more rare occurrence.

I find that there died of this disease in all the hospitals, regimental as well as general,

| | |
|---|---|
| in 1812 | 49 |
| 1813 | 158 |
| 1814, to 24th of June, | 72 |
| | 279 |

which, of the total mortality, excluding that from wounds, is a small proportion, and very widely different indeed from what I have found to be the case in the army in England. Under ordinary circumstances, and when no epidemic or contagious disease prevailed, I found, in England, the deaths from consumption to

2 amount amount to one-fifth, one-fourth, and, in some regiments, even as high as one half, of the whole mortality.

The expatriating phthisical patients to the milder southern climates, particularly to that of Portugal, has, for many years, been a highly-sanctioned practice in England. On going to Portugal, I was desirous to obtain information on this subject, and particularly after reading the excellent paper of Dr. Wells in the 3d vol. of the Transactions of a Society for the Improvement of Medical and Chirurgical Knowledge. I circulated a series of queries on the subject, and the sum of the answers from the principal medical officers at the stations in Portugal where we had hospitals is as follows:—In the period included, from the 25th June to the 24th September, 1812, it appears, that the cases of phthisis at six hospital stations amount to 54; of remittent and intermittent fever to 1880: therefore, the proportion of phthisis pulmonalis to remittent and intermittent fever is as 1 to 35.

The origin of the greater part of the cases is not distinctly stated; but, of 16 treated at Lisbon, 9 evidently originated in England, and 7 in Portugal, sent from different hospital stations to Lisbon.

It clearly appears, that, in the early stage of consumption, that is to say, when suppuration and ulceration have not yet taken place, the disease is checked by the climate of the Peninsula; but that, when suppuration and ulceration have taken place, it runs even a more rapid progress than in England; and I have made the same remark, in regard to the East and West Indies.

Of 30 cases of phthisis, 14 died, and one was not likely to recover; and the remainder continued in hospital to the end of the quarter. Of 24 at Coimbra, the proportion of deaths is not stated; but it is stated, that the disease in general proceeded rapidly to a fatal termination. The progress of phthisis appeared to be accelerated by attacks of intermittent fever, and the phthisical predisposition did not appear to confer any exemption from the attack of that disease.

I repeated my queries, and got the results of another quarter's inquiry, and I found that in this period, at Alta de Chaô, in Estremadura,

| | | | |
|---|---|---|---|
| 4 cases of phthisis appeared, | 2 of which originated at Alta de Chaô, | | |
| 7 cases of remittent fever, | 5 | Do. | Do. |
| 526 cases of ague, | 192 | Do. | Do. |

At Castello Branco, a very high and dry situation in Beira,

| | | | |
|---|---|---|---|
| Of 171 cases of continued fever, | 17 | originated there. |
| 169 | intermittent | 38 | Do. |

No case of phthisis.

At Lisbon,

3 cases of phthisis admitted, none of them originated at Lisbon.
31 remittent and intermittent fever; 17 originated at Lisbon.

At Santarem there appeared, during this period, 10 well-marked cases of phthisis, and 666 cases of intermittent fever.

At

At Celorico,
2 cases of phthisis,
50 remittent, and
66 intermittent fever.

2d division of the army, about 6,500 strong:
4 cases of phthisis pulmonalis admitted,
551 remittent fever,
1566 intermittent do.

On the whole, the result of much inquiry was, that phthisis and scrofula, although occasionally occurring, are rare diseases in Portugal. There seems to be but little predisposition in the natives, or in those some time resident in Portugal, to inflammatory and pulmonic complaints; and the latter seems to be less violent and more easily curable than in England. It would appear, that persons coming to Portugal or Spain, and, I may add, to part of France, from colder climates, with only a predisposition to phthisis, with tubercles in the lungs in a quiescent state, or not actually suppurating, do receive some benefit from the sea voyage or change of air, or rather that the disease does not make any progress. When, however, suppuration and ulceration have taken place, the result is almost always as fatal, and in as short a time, as if the experiment had not been tried of change of climate.

Dr. Fergusson, Dr. Somers, and the late Dr. Gray, resided each of them several years at Lisbon, and saw most of the phthisical cases sent there from England, particularly Dr. Somers, who resided there four years as a practising physician; and it is the result of the experience of these gentlemen, that, though the cough abated of its violence, as might naturally be expected from the milder temperature, that patients seldom received any other sensible alleviation. Dr. Somers informed me, that he never witnessed a complete recovery; insomuch, that, for the last two years, he uniformly set down the cases for return to their native country, resting assuredly some hope, in favourable seasons, on the probable efficacy of the voyage. He informed me, that he had never seen the genuine *British phthisis** originate in Portugal.

When I was at Montpellier, I learned from the professional gentlemen there, that the result of their experience on patients sent thither in these stages of phthisis, was nearly what I have stated to have been in Lisbon. They further informed me, that the high and very dry air of that place was particularly unfavourable to some species of the disease; and that not a few cases originated in that country.

The situation of Montpellier is singular: it will be recollected that it is intermediate between the Pyrenees and Alps; both are to be seen from the beautiful public gardens of the town. The vicinity of the mountains to the north of Montpellier renders the climate in winter and spring very changeable; and this is found to be a cause of much mischief to phthisical patients. Those cases which

* See our Account of the Climate of Madeira, vol. v. p. 307.

which originated in the country were generally from the neighbourhood of Montpellier. There are other situations that appear better for the consumptive than Montpellier, viz. between that place and Nismes, and near the banks of the Garonne, from Toulouse to Bourdeaux. Lisbon has many disadvantages for the phthisical, from the vicinity of a mountainous country and the neighbouring sea, and from the total want of protection in all the houses against change of temperature.

The result of our experience in Walcheren was, that the air was, in general, favourable to pulmonic complaints. A physician, from whom I have derived a good deal of the information which I now submit to the Society, in that island got entirely free of an asthma, under which he had laboured for many years of his life.

The appearance of Dr. Woolcombe's work excited my attention to this disease, as it appeared in the troops stationed in Hampshire, Somersetshire, Dorsetshire, and the neighbouring counties then under my medical inspection. The troops were mostly militia regiments, the greater part of them in garrison at Portsmouth, Bristol, or Weymouth. The surgeons had the best means of knowing the origin and every particular of the cases which occurred in their corps, and, at my request, paid much attention to them.

By a tabular statement, which, early in 1811, I drew out from the reports of these gentlemen, and my own examination of the cases, I find, that, from July 1808, to February 1811, I have a minute detail of 52 cases. The result is as follows:

31 died,
7 were cured,
7 were getting worse,
4 were better,
1 was discharged from his regiment,
2 went to live with their friends—fate not known.

Under the head Causes:

3 originated after continued fever,
18 - - Pneumonia,
7 - - Catarrh,
8 - - Hæmoptysis,
5 - - Rubeola,
3 - - Syphilis,
2 - - Asthma,
1 - - Scrofula,
3 from hereditary disposition,
1 from playing on wind instruments,
1 had obstruction of the abdominal viscera.

Of these men,

18 were labourers before they became soldiers,
13 were weavers,
5 Shoemakers,
3 Tailors,

2 Gardeners,

2 Gardeners,
1 Flax-dresser,
1 Clothier,
1 Hatter,
1 Needle-pointer,
1 Butcher,
1 Nailer,
1 Blacksmith,
1 Schoolmaster,
2 Unknown.

| | | | |
|---|---|---|---|
| 8 | of them occurred in the | | Lancashire Militia, |
| 7 | - | - | 8th Veteran Battalion, |
| 6 | - | - | Inverness Militia, |
| 6 | - | - | Sussex do. |
| 5 | - | - | Worcester do. |
| 4 | - | - | North Gloucester, |
| 3 | - | - | East Suffolk, |
| 3 | - | - | 1st York, |
| 2 | - | - | East Middlesex, |
| 2 | - | - | Glamorgan, |
| 3 | - | - | North Hants. |
| 1 | - | - | Northampton, |
| 1 | - | - | Pembroke, |
| 1 | - | - | Oxford, |
| 1 | - | - | Dorset, |
| 1 | - | - | 11th light dragoons. |

It is observable, that none of these cases occurred in the regiments of the marshy counties in which intermittent fevers are endemic.

---

